# Prolonged Severe Watery Diarrhea in a Long-Term Myeloma Survivor: An Unforeseen Infection with *Cystoisospora belli*

**DOI:** 10.4274/tjh.galenos.2020.2020.0414

**Published:** 2021-06-01

**Authors:** Tarık Onur Tiryaki, Kadir Uluç Anıl, Melek Büyük, Ahmet Yasir Yıldırım, Alp Atasoy, Aslı Çiftçibaşı Örmeci, Sevgi Kalayoğlu Beşışık

**Affiliations:** 1 İstanbul University İstanbul Medical Faculty, Department of Internal Medicine, Division of Hematology, İstanbul, Turkey; 2 İstanbul University İstanbul Medical Faculty, Department of Internal Medicine, İstanbul, Turkey; 3 İstanbul University İstanbul Medical Faculty, Department of Pathology, İstanbul, Turkey; 4 İstanbul University İstanbul Medical Faculty, Department of Internal Medicine, Division of Gastroenterohepatology, İstanbul, Turkey

**Keywords:** Cystoisospora belli, Prolonged watery diarrhea, Multiple myeloma

## To the Editor,

The parasite *Cystoisospora belli* (formerly known as *Isospora belli*), referred to as coccidian, infects the epithelial cells of the small intestine and is one of the least common intestinal coccidia that infect humans. Infections have been sporadically reported in a wide variety of immunocompromised patients, including patients with concurrent Hodgkin’s disease, non-Hodgkin’s lymphoma, and acute lymphoblastic leukemia, and can sometimes be fulminant in immunocompromised patients [1,2]. Chronic diarrhea is the major clinical manifestation, sometimes associated with headache, fever, malaise, abdominal pain, vomiting, dehydration, and weight loss. Extraintestinal infections with tissue cyst-like stages have been observed in the lymph nodes, liver, and spleen of patients with AIDS. Infections of immunosuppressed patients with *C. belli* have been reported in association with viral infections other than HIV, especially human T-cell leukemia virus type 1 (HTLV-1) [3].

Multiple myeloma (MM)-related immunodeficiency involves B-cell dysfunction, such as hypogammaglobulinemia, as well as T-cell, dendritic cell, and NK-cell abnormalities [4,5,6,7]. In addition to the disease-related inherent immunodeficiency, some studies have described a changing spectrum of infections in MM, possibly related to the more intensive or immunomodulating treatment approaches of recent years. Among MM cases, infections are a significant cause of morbidity and a leading cause of death. We present here a case of *C. belli* infection in a MM patient who developed persistent diarrhea in the late responsive period.

**Figure 1:**
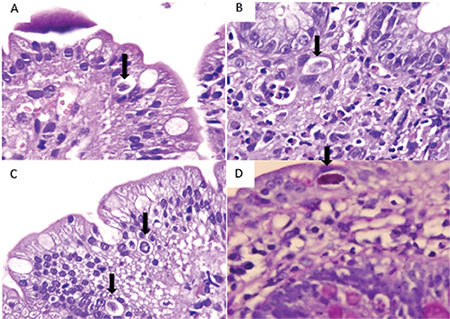
Sections of the duodenum (H&E stain, 1000^x^). *Cystoisospora belli* is present inside the epithelium (arrows) with a halo around it **(A, B, C)**. There are also many eosinophils, neutrophils, and lymphocytes in the lamina propria **(B)**. Pink granular staining of parasite with periodic acid-Schiff histochemical staining **(D)** (PAS, 1000^x^).

A 66-year-old male patient was diagnosed as having immunoglobulin (Ig) G kappa-type MM of Durie-Salmon stage IIIA in 2007. He underwent autologous stem cell transplantation (ASCT) following high-dose melphalan (200 mg/m^2^) after second-line treatment. A bortezomib-based regimen was used again due to progression in the first year after ASCT. A second ASCT was performed for consolidation, this time followed by lenalidomide maintenance. He achieved complete response, but at the 24^th^ month of maintenance, he relapsed. Pomalidomide, cyclophosphamide, and dexamethasone were started. Treatment regimens and disease characteristics are summarized in Table 1. In the 4^th^ month of this treatment, he developed severe watery diarrhea with abdominal pain, which became persistent despite supportive measures. MM disease status showed biochemical remission. Infectious causes were excluded by variable tests including ova and parasite screening. Acute phase markers were not remarkable. Metronidazole (500 mg orally, every 8 h for 5 days) and ciprofloxacin (500 mg orally, b.i.d.) were started empirically. There was no fat, blood, or leukocytes in the stool. Accompanying viral infection was not detected in our patient, such as HIV or HTLV-1. Anti-tissue transglutaminase IgA and anti-gliadin IgA antibodies were negative. Thyroid hormone levels, vasoactive intestinal peptide, and urine 5-HIAA levels were within normal ranges. Gastroscopy showed non-erosive antral gastritis and edematous and erythematous duodenum. He continuously lost weight and became pale, but had no fever. There was no gross pathology in colonoscopy except distinct thin vascular structures. Multiple biopsies were obtained randomly to evaluate amyloidosis. Contrast-enhanced abdominal CT did not reveal any abnormality. Endoscopic biopsy samples demonstrated mild duodenitis and colitis characterized by increased numbers of plasma cells and lymphocytes but no villous atrophy or crypt hyperplasia (Figure 1B). Amyloid staining proved to be negative. No inclusion bodies were found, pointing to viral infection. In the duodenal epithelium, beneath the nuclei, a different image was striking. There were PAS-stained granules (Figure 1D) and oocysts were identified, which were consistent with *C. belli*. Several developmental stages of *C. belli* parasites in duodenal epithelial cells were identified (Figures 1A-1C). Treatment with trimethoprim (TMP)-sulfamethoxazole (160 mg of trimethoprim and 800 mg of sulfamethoxazole [STX]) as one double-strength tablet b.i.d. orally for 10 days improved the clinical picture dramatically and the patient began to gain weight. His MM status is still CR and he is on TMP/STX prophylaxis.

*Cystoisospora belli* infections are essentially cosmopolitan in distribution but are more common in tropical and subtropical regions [8]. Clinical presentation may mimic inflammatory bowel disease and irritable bowel syndrome. In immunocompromised patients, infection is often severe, with a secretory-like diarrhea that may lead to dehydration and require hospitalization, sometimes associated with fever and weight loss [6,8]. For our patient, the infection presentation was chronic watery diarrhea, which contributed to severe weight loss. *C. belli* diagnosis is performed by detection of the oocysts in stool samples by direct microscopy or by modified Ziehl-Neelsen staining methods and autofluorescence technique. There is no reported serological test at present [3]. In our case, direct microscopy did not capture oocysts. Oocysts may rarely be detected in gastrointestinal epithelium or bile samples. However, careful examination of intestinal biopsy samples has helped detect PAS-positive granules and oocysts [8]. In Turkey, *C. belli* infection has been reported sporadically. Cystoisosporiasis can be prevented with adequate sanitation, measures to protect food and water supplies, and increased public awareness of the means of transmission [8,9]. Immunocompetent hosts generally respond very rapidly and tend to improve in 2-3 days with antiparasitic therapy [9]. Immunocompromised hosts also respond well, though less rapidly. However, these individuals relapse at a high rate once therapy is stopped. Such patients may need life-long suppressive treatment with TMP-STX. Intravenous administration of TMP-STX is more effective when the disease is extraintestinal. Cessation of diarrhea and the disappearance of *C. belli* oocysts from stool samples are the endpoints for monitoring therapy.

Increased susceptibility to bacterial infections is a common manifestation of MM, arising mainly from a defect in humoral immunity and associated with major morbidity and mortality [4,7,10]. The risk is highest in the first three months after diagnosis and decreases with treatment. Rarely, opportunistic infections may also be seen mainly in the late period [6,11]. Parasitic infections are very uncommon among MM patients [11]. In MM diarrhea points mainly to infection in acute or chronic form. Other intestinal or hormonal diseases should be excluded. AL amyloidosis is a plasma cell disease-related reason. We wish to attract attention to this rare case of parasitic infection in MM. To our knowledge, this the first case of a patient with MM with *C. belli* infection.

## Figures and Tables

**Table 1:**
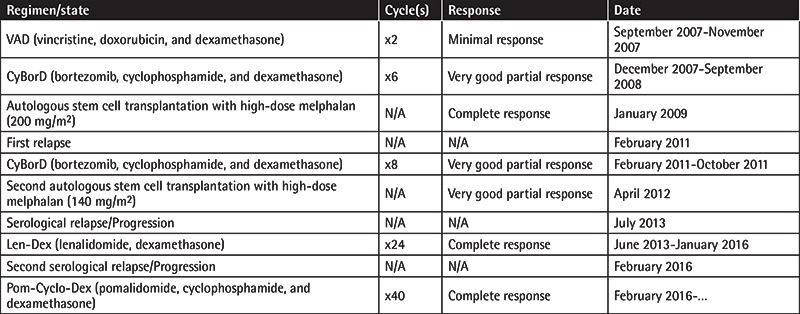
Treatment regimens and disease characteristics.
